# Don't Stop Belonging: Associations Between National Narcissism, Self‐Esteem and Optimism in Social Belonging

**DOI:** 10.1002/ijop.70184

**Published:** 2026-02-08

**Authors:** Cristian Ramos‐Vera, Iraklis Grigoropoulos, Luis Hualparuca‐Olivera, Roger Angulo Salas, Dritjon Gruda

**Affiliations:** ^1^ Universidad Cesar Vallejo Lima Peru; ^2^ International Hellenic University Thessaloniki Greece; ^3^ Escuela de Psicología Universidad Continental Huancayo Peru; ^4^ Universidad Católica Sedes Sapientiae Lima Peru; ^5^ Universidade Católica Portuguesa, Católica Porto Business School and Research Centre in Management and Economics Porto Portugal; ^6^ Maynooth University School of Business Maynooth Ireland

**Keywords:** belonging, individual differences, national narcissism, optimism, self‐esteem

## Abstract

This study examines the relationship between national narcissism, a defensive form of collective pride contingent on external validation and social belonging. Drawing on self‐determination theory and social identity theory, we investigate the mediating role of self‐esteem in this relationship, potentially transforming belonging from a defensive, compensatory strategy into a genuine desire for connection. We also explore whether optimism and sex moderate this mediated relationship. In a sample of 44,407 participants across 51 countries, we find that self‐esteem mediates the association between national narcissism and social belonging, with higher self‐esteem reducing defensive reliance on external validation. Both optimism and sex further moderate these effects: optimism buffered against the negative impact of national narcissism, while men and women exhibited distinct pathways in translating national narcissism into belonging. Our findings demonstrate that self‐esteem determines whether national narcissism fosters insecure or more authentic forms of belonging. Moreover, both dispositional outlook (optimism) and demographic factors (sex) significantly influence the extent to which national narcissism shapes social connection.

## 1 Introduction

Narcissism has long been characterised by an instrumental view of social relationships, in which the primary aim is to secure personal advantages, such as status, rather than to foster close, meaningful bonds (Gruda et al. 2021, 2022; Gruda and Hanges 2023). Yet, empirical findings have been inconsistent. Some studies report negative associations between narcissism and belonging‐related constructs (Ojanen et al. 2012), others suggest no significant relationship (Findley and Ojanen 2013) or even a small positive correlation (Zeigler‐Hill et al. 2019). Whether such inconsistent findings extend to national narcissism (i.e., a form of collective narcissism marked by an inflated yet fragile belief in one's nation's superiority) remains insufficiently understood.

In this paper, we investigate the extent to which national narcissism is associated with social belongingness and whether self‐esteem influences this relationship. Scholars have argued that national narcissism often originates from unmet personal needs for belongingness or recognition (Eker and Cichocka 2024). The mediating role of self‐esteem is critical here: when self‐esteem is sufficiently nurtured, individuals may feel less dependent on group‐based validation for maintaining a sense of worth. Additionally, we examine whether individual differences—specifically optimism and sex—moderate this relationship.

### 1.1 Belongingness and National Narcissism

A strong sense of belonging is frequently considered essential to self‐esteem, as it provides a sense of security rooted in group acceptance. Prior research on conventional forms of ingroup identification has shown that group membership can fulfil a range of needs, including belongingness and self‐esteem (Greenaway et al. 2015). Social Identity Theory contends that people derive part of their self‐concept from group memberships, which become vital sources of self‐esteem and identity (Tajfel and Turner 1979).

In national group contexts, national identity can be a key component of one's self‐concept. National narcissism involves an exaggerated yet insecure conviction in one's nation's superiority, along with an expectation of external validation of this conviction. Such national pride may serve as a source of self‐worth by fulfilling a need for group‐based belonging, offering individuals a stable, idealised identity linked to their national group.

We propose that high national narcissism often indicates underlying insecurities, including a desire for external validation of self‐worth through membership in a prestigious group. This reliance on the group may undermine authentic social belonging by intensifying defensiveness, particularly among individuals with low self‐esteem (Osborne et al. 2019). As self‐esteem increases, highly (national) narcissistic individuals may develop a more secure self‐concept, enabling them to seek positive social connections rather than perpetual reassurance. We hypothesise:

*National narcissism is negatively associated with self‐esteem, which in turn is positively related to social belonging, such that the relationship between national narcissism and social belonging operates indirectly through self‐esteem*.

### 1.2 The Moderating Role of Optimism and Sex

Optimism, defined as a generalised expectancy that favourable outcomes will occur (Scheier and Carver 1985), may negatively moderate the indirect relationship between national narcissism and social belonging via self‐esteem. By enhancing social interactions and perceived support, high optimism correlates positively with a sense of belonging. Consequently, for optimistic individuals, national narcissism may exert a weaker influence on both self‐esteem and social belonging, as they tend to have a built‐in sense of security and rely less on external validation through their ingroup. Thus, optimism could buffer, or weaken, the mediated path from national narcissism to social belonging. We hypothesise:

*Optimism moderates the relationship between national narcissism and social belonging, such that the relationship is stronger (weaker) when optimism is low (high)*.

Sex may also operate as a moderator. de Golec Zavala and Keenan (2024) noted sex differences in national and collective narcissism, suggesting that men and women experience national pride differently, especially concerning egalitarian social aims. For men, national narcissism aligns with rejecting egalitarian ideologies and preserving existing hierarchies. In contrast, for women, it can entail internalising patriarchal values, thereby reducing egalitarian views in favour of national identity. We posit that for highly nationally narcissistic men, lower self‐esteem may yield a preoccupation with status preservation, resulting in defensive rather than authentic social belonging. Higher self‐esteem might mitigate this defensiveness. For women, elevated self‐esteem may counteract the adverse effects of national narcissism on belonging by fostering more inclusive values. Conversely, lower self‐esteem could reinforce patriarchal norms linked to national identity, leading to less genuine belonging. We hypothesise the following:

*Sex moderates the relationship among national narcissism, self‐esteem and sense of belonging. Specifically, we expect the indirect effect of national narcissism on social belonging via self‐esteem to differ between men and women*.

## 2 Method

### 2.1 Sample and Procedures

To conduct this study, we utilised a publicly available dataset of 44,407 participants (*M* = 43.07, SD = 16.07; 51.7% female) from 51 countries, part of the Social & Moral Psychology of COVID‐19 project (Azevedo et al. 2023). Data were collected between 22 April and 3 June 2020. The full dataset included 51,489 individuals; however, we excluded observations with incomplete responses or non‐contributing data. Given that this study involves the secondary analysis of a pre‐existing dataset and the specific hypotheses were not preregistered, the present investigation should be interpreted as exploratory. Respective files and scripts are publicly accessible at https://osf.io/z58hj/.

### 2.2 Measures

#### 2.2.1 Social Belonging (SB)

We used the four‐item General Belongingness Scale (GBS) to measure social belonging (Malone et al. 2012; *ω* = 0.78). Example items: ‘When I am with other people, I feel included’ and ‘I feel connected to others’.

#### 2.2.2 National Narcissism (NrcN)

The Collective Narcissism Scale measures the extent to which an individual believes their country is superior and exhibits an inflated, irrational sense of national pride (de Golec Zavala et al. 2009). Two items were used (*ω* = 0.78), including ‘[My national group] deserves special treatment’ and ‘Not many people seem to fully understand the importance of [my national group]’.

#### 2.2.3 Self‐Esteem (SE)

Self‐esteem was measured using the Single‐Item Self‐Esteem Scale (SISE; Robins et al. 2001). The respective item is: ‘I have high self‐esteem’. This measure has established itself as a valid alternative to longer self‐esteem scales in specific contexts and has been utilised in several previous studies (e.g., Pavlović et al. 2022).

#### 2.2.4 Trait Optimism (TrO)

The Life Orientation Test‐Revised (LOT‐R; Scheier et al. 1994) was used to measure trait optimism. The 10‐item scale (*ω* = 0.83) measures a person's tendency to see the positive side and remain optimistic in the face of adversity. Example items include ‘As a person, I am optimistic about my future’ and ‘In general, I expect more good things to happen to me than bad things’.

### 2.3 Data Analysis

First, we assessed the mediation of self‐esteem (SE) in the relationship between national narcissism (NrN) and social belonging (SB), considering both direct and indirect effects. The mediating relationship (national narcissism → self‐esteem → social belonging) was analysed at three different levels of moderators. The statistical significance of the pathways, along with their moderating and mediating effects, was assessed using the Bootstrap percentile method, which involved 5000 resamples to estimate a 95% confidence interval (Hayes 2022). An effect was considered statistically significant if the confidence interval excluded zero. All analyses were performed using Jamovi software (version 2.3.21). We used the GLM Mediation Model module (JAMOVI‐ Advanced Mediation Models) to analyse moderated mediation.

Subsequently, the moderating role of the level of optimism (TrO) and sex (SEX) was analysed. Since the moderating variable of sex was taken into account, 160 participants who did not respond to this sociodemographic category were excluded from the moderated mediation analysis. To assess moderation, three levels of TrO were established: low (mean − 1 SD), medium (Mean) and high (mean + 1 SD). Sex (1 = male, 2 = female) was incorporated as a moderating variable.

## 3 Results

Descriptive statistics and respective correlation coefficients are shown in Table 1.

**TABLE 1:** Descriptive analysis.

	Mean	SD	Skew	Kurtosis	NarcN	TrO	SB	SE
NarcN	0.09	0.09	0.49	−1.68	—			
TrO	0.11	0.15	0.46	−1.63	0.16	—		
SB	0.11	0.15	−0.13	−1.73	0.14	0.31	—	
SE	0.12	0.12	0.48	−1.57	0.18	0.32	0.23	—

Abbreviations: NarcN, national narcissism; *R* ^2^, explained variance; SB, social belonging; SE, self‐esteem; TrO, trait optimism.

Moderation analyses reveal three significant interactions that reflect how certain variables moderate the relationships between national narcissism, self‐esteem and social belonging (Table 2).

**TABLE 2:** Moderation effects (interactions).

Interaction	*B*	Lower	Upper	*β*	*p*
Sex * SE ⇒ SB	−0.096	−0.145	−0.047	−0.044	< 0.001
TRO * NARCN ⇒ SE	−0.001	−0.001	0.000	−0.009	0.039
TRO * SE ⇒ SB	−0.013	−0.018	−0.008	−0.049	< 0.001

Abbreviations: *β*, standardised estimator; *B*, non‐standardised estimator; NARCN, national narcissism; *p*, statistical significance; SB, social belonging; SE, self‐esteem; Sex, sex; TRO, optimism.

A significant interaction between sex and self‐esteem was found (*β* = −0.044, *p* < 0.001), indicating that the relationship between self‐esteem and social belonging varies by sex. As self‐esteem increases, its influence on social belonging is lower in men compared to women.

Optimism also moderated the relationship between narcissism and self‐esteem (*β* = −0.009, *p* = 0.039). Specifically, in individuals with higher optimism, the impact of narcissism on self‐esteem is lower. Optimism also moderated the relationship between self‐esteem and social belonging (*β* = −0.049, *p* < 0.001). In highly optimistic individuals, the relationship between self‐esteem and social belonging is weaker.

Next, indirect, direct and total effects are reported for different conditions of the moderation. TrO was tested at three levels: low (mean − 1 SD), medium (Mean) and high (mean + 1 SD). Results in the descriptive text below relate to the medium level (Table 3).

**TABLE 3:** Conditional mediation.

Moderator levels		Type	Effect	*B*	95% CI (a)		*β*	*p*
TRO	Sex				Lower	Upper		
Mean − 1·SD	Average	Indirect	NARCN ⇒ SE ⇒ SB	0.032	0.029	0.035	0.036	< 0.001
Mean − 1·SD	Average	Component	NARCN ⇒ SE	0.039	0.036	0.043	0.135	< 0.001
Mean − 1·SD	Average		SE ⇒ SB	0.818	0.79	0.846	0.263	< 0.001
Mean − 1·SD	Average	Direct	NARCN ⇒ SB	0.027	0.02	0.035	0.03	< 0.001
Mean − 1·SD	Average	Total	NARCN ⇒ SB	0.054	0.047	0.062	0.06	< 0.001
Mean − 1·SD	1	Indirect	NARCN ⇒ SE ⇒ SB	0.034	0.031	0.037	0.038	< 0.001
Mean − 1·SD	1	Component	NARCN ⇒ SE	0.039	0.036	0.043	0.135	< 0.001
Mean − 1·SD	1		SE ⇒ SB	0.866	0.838	0.894	0.277	< 0.001
Mean − 1·SD	1	Direct	NARCN ⇒ SB	0.027	0.02	0.035	0.03	< 0.001
Mean − 1·SD	1	Total	NARCN ⇒ SB	0.054	0.047	0.062	0.06	< 0.001
Mean − 1·SD	2	Indirect	NARCN ⇒ SE ⇒ SB	0.03	0.027	0.033	0.034	< 0.001
Mean − 1·SD	2	Component	NARCN ⇒ SE	0.039	0.036	0.043	0.135	< 0.001
Mean − 1·SD	2		SE ⇒ SB	0.77	0.742	0.798	0.248	< 0.001
Mean − 1·SD	2	Direct	NARCN ⇒ SB	0.027	0.02	0.035	0.03	< 0.001
Mean − 1·SD	2	Total	NARCN ⇒ SB	0.054	0.047	0.062	0.06	< 0.001
Mean	Average	Indirect	NARCN ⇒ SE ⇒ SB	0.028	0.026	0.03	0.031	< 0.001
Mean	Average	Component	NARCN ⇒ SE	0.037	0.035	0.04	0.127	< 0.001
Mean	Average		SE ⇒ SB	0.761	0.733	0.789	0.245	< 0.001
Mean	Average	Direct	NARCN ⇒ SB	0.027	0.02	0.035	0.03	< 0.001
Mean	Average	Total	NARCN ⇒ SB	0.054	0.047	0.062	0.06	< 0.001
Mean	1	Indirect	NARCN ⇒ SE ⇒ SB	0.03	0.028	0.032	0.033	< 0.001
Mean	1	Component	NARCN ⇒ SE	0.037	0.035	0.04	0.127	< 0.001
Mean	1		SE ⇒ SB	0.809	0.781	0.837	0.26	< 0.001
Mean	1	Direct	NARCN ⇒ SB	0.027	0.02	0.035	0.03	< 0.001
Mean	1	Total	NARCN ⇒ SB	0.054	0.047	0.062	0.06	< 0.001
Mean	2	Indirect	NARCN ⇒ SE ⇒ SB	0.026	0.024	0.028	0.029	< 0.001
Mean	2	Component	NARCN ⇒ SE	0.037	0.035	0.04	0.127	< 0.001
Mean	2		SE ⇒ SB	0.713	0.685	0.741	0.23	< 0.001
Mean	2	Direct	NARCN ⇒ SB	0.027	0.02	0.035	0.03	< 0.001
Mean	2	Total	NARCN ⇒ SB	0.054	0.047	0.062	0.06	< 0.001
Mean + 1·SD	Average	Indirect	NARCN ⇒ SE ⇒ SB	0.024	0.022	0.027	0.027	< 0.001
Mean + 1·SD	Average	Component	NARCN ⇒ SE	0.035	0.032	0.038	0.119	< 0.001
Mean + 1·SD	Average		SE ⇒ SB	0.704	0.676	0.732	0.227	< 0.001
Mean + 1·SD	Average	Direct	NARCN ⇒ SB	0.027	0.02	0.035	0.03	< 0.001
Mean + 1·SD	Average	Total	NARCN ⇒ SB	0.054	0.047	0.062	0.06	< 0.001
Mean + 1·SD	1	Indirect	NARCN ⇒ SE ⇒ SB	0.026	0.024	0.029	0.029	< 0.001
Mean + 1·SD	1	Component	NARCN ⇒ SE	0.035	0.032	0.038	0.119	< 0.001
Mean + 1·SD	1		SE ⇒ SB	0.752	0.724	0.78	0.242	< 0.001
Mean + 1·SD	1	Direct	NARCN ⇒ SB	0.027	0.02	0.035	0.03	< 0.001
Mean + 1·SD	1	Total	NARCN ⇒ SB	0.054	0.047	0.062	0.06	< 0.001
Mean + 1·SD	2	Indirect	NARCN ⇒ SE ⇒ SB	0.023	0.02	0.025	0.025	< 0.001
Mean + 1·SD	2	Component	NARCN ⇒ SE	0.035	0.032	0.038	0.119	< 0.001
Mean + 1·SD	2		SE ⇒ SB	0.656	0.628	0.684	0.212	< 0.001
Mean + 1·SD	2	Direct	NARCN ⇒ SB	0.027	0.02	0.035	0.03	< 0.001
Mean + 1·SD	2	Total	NARCN ⇒ SB	0.054	0.047	0.062	0.06	< 0.001

Abbreviations: *β*, standardised estimator; *B*, non‐standardised estimator; CI, confidence interval; NARCN, national narcissism; *p*, statistical significance; SB, social belonging; SE, self‐esteem; Sex, sex (1 = males; 2 = female); TRO, optimism.

The indirect effect of national narcissism on social belonging via self‐esteem was significant across all conditions (supporting H1), though the effects were minimal. Consistent with H2 and H3, the magnitude of this indirect effect varied by optimism and sex (see Table 3). Specifically, as optimism increased, the indirect effect weakened. Furthermore, the indirect effect was consistently slightly stronger for men than for women across all levels of optimism (e.g., at Mean TrO: Men *β* = 0.033; Women *β* = 0.029). The direct effect of narcissism on social belonging remained significant across all conditions.

## 4 Discussion

The present study expands on growing evidence that national narcissism may be linked to the fundamental psychological need for social belonging (e.g., Eker and Cichocka 2024; de Golec Zavala et al. 2009). Consistent with Social Identity Theory (Tajfel and Turner 1979) and prior findings on collective narcissism (Eker and Cichocka 2024), our results demonstrate that self‐esteem serves as a mediating factor between national narcissism and social belonging. Individuals with higher levels of national narcissism often seek validation through their national group, attempting to offset insecurities or unmet personal demands. However, when self‐esteem is strengthened, the defensive quest for external validation may decrease, making room for more stable and genuine social connections. This aligns with recent work underscoring the importance of self‐esteem in buffering against defensive or compensatory social identifications (Eker and Cichocka 2024) and corroborates the notion that belonging need not be strictly reactive or defensive (Allen et al. 2020).

The moderating effect of optimism further refines our understanding of these dynamics. Rooted in the foundational work of Scheier and Carver (1985), optimism has been consistently associated with positive coping strategies and enhanced social support. Our findings align with prior research, which demonstrates that optimistic individuals tend to require less external validation to maintain their self‐esteem (Duy and Yıldız 2017), thereby diminishing the influence of national narcissism on both self‐esteem and social belonging. In this sense, optimism can operate as a psychological buffer, reducing defensive tendencies and enabling a more constructive pursuit of belonging needs.

Sex differences also emerged as a meaningful aspect of how national narcissism translates into social belonging via self‐esteem. Echoing de Golec Zavala and Keenan (2024), our study suggests that men may use national narcissism to preserve or enhance their social status, potentially relegating belonging needs to a lower priority when their self‐esteem is threatened. Women, on the other hand, may experience these processes through different pathways, sometimes internalising patriarchal norms within their national identity when their self‐esteem is low, or aligning with more inclusive values when their self‐esteem is high. Taken together, these findings refine earlier work by illustrating how national narcissism's impact on belonging is not monolithic but instead contingent upon both personality variables (optimism) and demographic factors (sex).

In sum, our results offer a nuanced picture in which national narcissism, self‐esteem, optimism and sex coalesce to shape individuals' social belongingness. This aligns with prior theoretical perspectives suggesting that collective and national identities fulfil needs for belongingness and self‐esteem (Greenaway et al. 2015), but also highlights the complexity inherent in the distinction between defensive and genuine forms of social identity.

## 5 Limitations

Although our findings contribute to our understanding of the psychology of national narcissism and belonging, several significant limitations merit consideration. First, the study is limited by several measurement issues. National narcissism was measured with only two items, restricting the scope of the construct. Critically, the central mediator, self‐esteem, was measured with a single item (SISE). Furthermore, while our theoretical framework distinguishes between ‘defensive’ and ‘genuine’ belonging, the General Belongingness Scale measures only the *degree* of belonging, not its underlying motivation or quality. Consequently, our interpretation that high self‐esteem facilitates ‘genuine’ belonging is an inference derived from the interaction patterns, rather than a direct observation of belonging subtypes.

Second, while the massive sample size (*N* = 44,407) provides high statistical power, the effect sizes observed in this study were quite small (e.g., indirect effects around *β* = 0.03; moderation effects as low as *β* = −0.009). This suggests that while national narcissism and self‐esteem are relevant factors, they are likely just two among many variables that influence an individual's sense of social belonging. Readers should therefore interpret these findings with caution, focusing on the direction and reliability of the associations rather than their magnitude.

Third, our cross‐sectional design precludes definitive conclusions about directionality. The associations we observe between national narcissism, self‐esteem and belonging could reflect various causal pathways, including reverse causation or bidirectional relationships. Moreover, as noted by Maxwell et al. (2011), cross‐sectional mediation analyses can yield biased estimates of longitudinal parameters. Variables identified as mediators in cross‐sectional models may not function as such over time, and cross‐sectional estimates may overstate the existence or magnitude of mediation. Future experimental or longitudinal studies could more definitively test whether increases in self‐esteem indeed reduce defensiveness and thereby temper national narcissism's adverse effects on belonging.

Fourth, although our sample was drawn from 51 countries, the present analyses were conducted at the aggregate individual level and did not account for the nesting of participants within countries. Future research should employ multilevel modelling to partition variance into within‐country and between‐country components, thereby testing whether these psychological processes are robust across different cultural contexts.

## 6 Conclusion

The present study underscores the potential importance of self‐esteem as a mediating mechanism that influences whether national narcissism promotes or inhibits a genuine sense of social belonging. At the same time, optimism and sex serve as significant moderators, adding depth to existing theoretical frameworks on collective narcissism. Our findings tentatively clarify the conditions under which group‐based pride can support or undermine a core human need for belonging.

## Author Contributions

**Luis Hualparuca‐Olivera:** data curation, writing – review and editing. **Roger Angulo Salas:** writing – review and editing. **Cristian Ramos‐Vera:** conceptualization, investigation, methodology, formal analysis, writing – original draft. **Dritjon Gruda:** writing – review and editing. **Iraklis Grigoropoulos:** writing – original draft.

## Funding

The authors have no funding to report.

## Ethics Statement

The authors confirm that they have read and conformed to the ethical standards guidelines listed in the journal's author guidelines. This study utilised a publicly available, anonymised dataset collected by the Social & Moral Psychology of COVID‐19 project (Azevedo et al. 2023). The primary researchers of that project obtained ethical approval for the original data collection. As this manuscript involves the secondary analysis of de‐identified data, it was exempt from further institutional review board approval.

## Consent

Informed consent was obtained from all individual participants by the collectors of the original dataset (Azevedo et al. 2023).

## Conflicts of Interest

The authors declare no conflicts of interest.

## Data Availability

The authors confirm they have access to the original data on which this paper reports. The dataset analysed during the current study is publicly available in the Open Science Framework repository at https://osf.io/z58hj/.
